# Supplementary material for: Association of Total and Trimester-Specific Gestational Weight Gain Rate with Early Infancy Weight Status: A Prospective Birth Cohort Study in China
Source: Nutrients. 2019 Jan 28;11(2):280. doi: 10.3390/nu11020280 (PMC6413060; doi:10.3390/nu11020280)
Supplement: Supplementary file 1 [file nutrients-11-00280-s001.pdf]

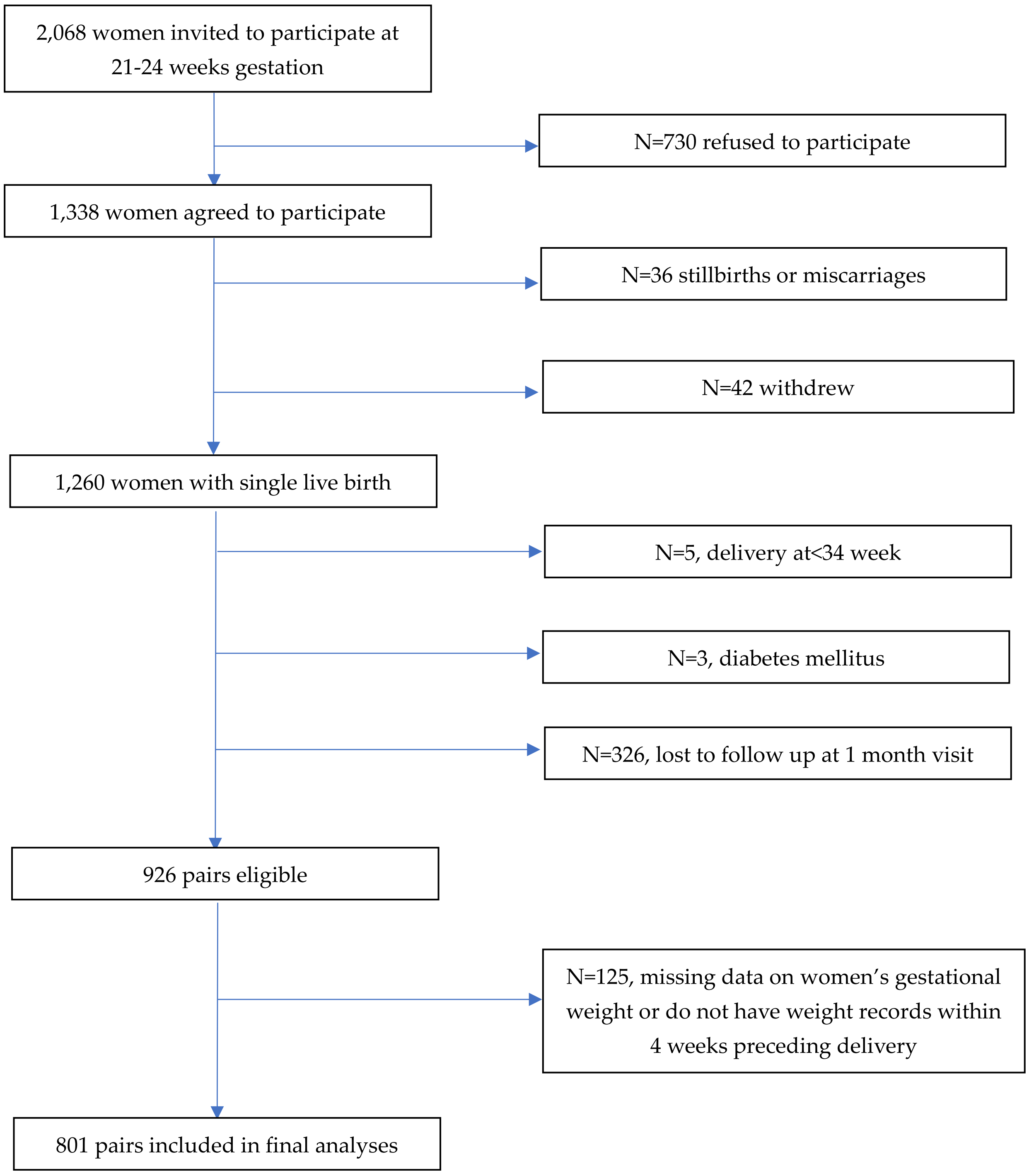

Figure S1. Study flow diagram.

Table S1. Associations of total and trimester-specific gestational weight gain rate with infant weight-for-length z-score from birth to 6 months using linear mixed effects model

	Total GWG rate (kg/wk)	1st & 2nd trimesters GWG rate (kg/wk)	3rd trimester GWG rate (kg/wk)
	β (95% CI) , (n=801)		
WFLZ			
Model 1	0.80 (0.29, 1.31) **	0.70 (0.22, 1.18) **	0.14 (-0.09, 0.36)
Model 2	0.96 (0.44, 1.47) ***	0.84 (0.36, 1.32) **	0.16 (-0.07, 0.38)
Model 3	0.96 (0.45, 1.47) ***	0.88 (0.40, 1.36) ***	0.14 (-0.08, 0.36) [a]

Model 1: adjusted for exact age of infants at each measurement

Model 2: Model 1 + pre-pregnancy body mass index

Model 3: Model 2 + maternal age, race, parity, education, household income, smoking status, paternal body mass index

[a]: Further adjust 1st & 2nd trimesters GWG rate, gestational diabetes

WFLZ, weight-for-length z-score; **: $p<0.01$, ***: $p<0.001$

Table S2. Associations of total and trimester-specific gestational weight gain rate with infant growth measures from birth to 6 months using linear mixed effects model, further adjusting for infant feeding style, television watching and secondhand smoking exposure at 1 month.

	Total GWG rate (kg/wk)	1st & 2nd trimesters GWG rate (kg/wk)	3rd trimester GWG rate (kg/wk) [a]
	β (95% CI), (n=663)		
BMIZ	1.32 (0.83, 1.82) ***	1.31 (0.85, 1.77) ***	0.08 (-0.14, 0.29)
WFLZ	0.95 (0.44, 1.46) ***	0.87 (0.39, 1.35) ***	0.14 (-0.09, 0.36)
WFAZ	1.20 (0.76, 1.63) ***	1.08 (0.68, 1.49) ***	0.07 (-0.12, 0.26)
LFAZ	0.51 (-0.04, 1.07)	0.41 (-0.11, 0.93)	-0.03 (-0.28, 0.21)

Adjusted for exact age of infants at each measurement, pre-pregnancy body mass index, maternal age, race, parity, education, household income, smoking status, paternal body mass index, infant feeding style at 1 month, infant television watching status at 1 month, second hand smoking exposure at 1 month.

[a]: Further adjust 1st & 2nd trimesters GWG rate, gestational diabetes

BMIZ, body mass index-for-age and sex z-score; WFAZ, weight-for-age z-score; LFAZ, length-for-age z-score; WFLZ, weight-for-length z-score; ***: p<0.001

Table S3. Associations of pre-pregnancy weight status with infant growth measures from birth to 6 months

	Pre-pregnancy BMI (kg/m^2)
BMIZ	β (95% CI), (n=801)
0 month	0.20 (0.07, 0.33)
1 month	0.20 (0.09, 0.31)
3 months	0.23 (0.11, 0.35)
6 months	0.24 (0.11, 0.38)

Adjusted for gestational weight gain rate, maternal age, race, parity, education, household income, smoking status, paternal body mass index.

BMI, body mass index; BMIZ, body mass index-for-age and sex z-score
